# Correlates of HIV, HBV, HCV and syphilis infections among prison inmates and officers in Ghana: A national multicenter study

**DOI:** 10.1186/1471-2334-8-33

**Published:** 2008-03-07

**Authors:** Andrew A Adjei, Henry B Armah, Foster Gbagbo, William K Ampofo, Isaac Boamah, Clement Adu-Gyamfi, Isaac Asare, Ian FA Hesse, George Mensah

**Affiliations:** 1 Department of Pathology, University of Ghana Medical School, College of Health Sciences, University of Ghana, Accra, Ghana; 2 Department of Virology, Noguchi Memorial Institute for Medical Research, College of Health Sciences, University of Ghana, Accra, Ghana; 3 Department of Microbiology, University of Ghana Medical School, College of Health Sciences, University of Ghana, Accra, Ghana; 4 Department of Medicine and Therapeutics, University of Ghana Medical School, College of Health Sciences, University of Ghana, Accra, Ghana; 5 Department of Community Health, University of Ghana Medical School, College of Health Sciences, University of Ghana, Accra, Ghana

## Abstract

**Background:**

Prisons are known to be high-risk environments for the spread of bloodborne and sexually transmitted infections. Prison officers are considered to have an intermittent exposure potential to bloodborne infectious diseases on the job, however there has been no studies on the prevalence of these infections in prison officers in Ghana.

**Methods:**

A national multicenter cross-sectional study was undertaken on correlates of human immunodeficiency virus (HIV), hepatitis B virus (HBV), hepatitis C virus (HCV), and syphilis infections in sample of prison inmates and officers from eight of ten regional central prisons in Ghana. A total of 1366 inmates and 445 officers were enrolled between May 2004 and December 2005. Subjects completed personal risk-factor questionnaire and provided blood specimens for unlinked anonymous testing for presence of antibodies to HIV, HCV and *Treponema pallidum*; and surface antigen of HBV (HBsAg). These data were analyzed using both univariate and multivariate techniques.

**Results:**

Almost 18% (1336) of 7652 eligible inmates and 21% (445) of 2139 eligible officers in eight study prisons took part. Median ages of inmates and officers were 36.5 years (range 16–84) and 38.1 years (range 25–59), respectively. Among inmates, HIV seroprevalence was 5.9%, syphilis seroprevalence was 16.5%, and 25.5% had HBsAg. Among officers tested, HIV seroprevalence was 4.9%, HCV seroprevalence was 18.7%, syphilis seroprevalence was 7.9%, and 11.7% had HBsAg. Independent determinants for HIV, HBV and syphilis infections among inmates were age between 17–46, being unmarried, being illiterate, female gender, being incarcerated for longer than median time served of 36 months, history of homosexuality, history of intravenous drug use, history of sharing syringes and drug paraphernalia, history of participation in paid sexual activity, and history of sexually transmitted diseases. Independent determinants for HIV, HBV, HCV and syphilis infections among officers were age between 25–46, fale gender, being unmarried, being employed in prison service for longer than median duration of employment of 10 years, and history of sexually transmitted diseases.

**Conclusion:**

The comparably higher prevalence of HIV, HBV, HCV and syphilis in prison inmates and officers in Ghana suggests probable occupational related transmission. The implementation of infection control practices and risk reduction programs targeted at prison inmates and officers in Ghana is urgently required to address this substantial exposure risk.

## Background

Prison populations are considered to be at high risk for bloodborne and sexually transmitted infections, such as the human immunodeficiency virus (HIV), hepatitis B virus (HBV), hepatitis C virus (HCV), and syphilis, due to the high proportion of intravenous drug users (IDU), commercial sex workers (CSW), men who have sex with men (MSM), and the homeless [1,2]. There is growing evidence that these infections have actually been transmitted to individuals while they were in prison [3-8], although there is also evidence that some had these infections before they were incarcerated. Numerous studies have estimated the occupational risk of exposure and infection with bloodborne pathogens for hospital-based and correctional healthcare workers (category I workers with regular or frequent exposure potential) [9-14], but similar data are sparse for prison officers not employed in healthcare delivery (PONEIHD, Category II workers with intermittent exposure potential). This is a concern not only because of high prevalence rates of bloodborne and sexually transmitted infectious diseases among prison inmates, but also because numerous challenges to the implementation of standard infection control practices in the correctional setting have been identified [15-17]. Additionally, although risk reduction recommendations to prevent the transmission of bloodborne pathogens in hospitals have been promulgated and evaluated, the degree of implementation and effectiveness of similar recommendations targeting correctional facilities remains largely unknown [18].

Data on the prevalence and risk factors for HIV, HBV, HCV and syphilis infections in prisoners are scanty in Africa. We recently reported a higher prevalence of these infections in inmates and correctional officers of 2 regional central prisons in Ghana compared to the general Ghanaian population [19]. The seroprevalence of HIV, HBV, HCV and syphilis in prisoners was 19.2%, 17.4%, 19.2% and 11%, respectively, and comparable to 8.5%, 3.7%, 23.2% and 4.9%, respectively among the prison officers in these 2 sampled prisons in Ghana [19]. A similar seroepidemiology survey in prisoners and prison officers in Italy reported comparable HbsAg carrier rate of 6.7% in prisoners and 6.6% in prison officers [20]. A more recent similar study in Greece reported a higher HbsAg carrier rate of 20.0% in prison officers compared to a rate of 13.3% in prisoners, but a lower HCV seroprevalence of 5.0% in prison officers compared to a seroprevalence of 15.5% in prisoners [21]. We recently demonstrated that the higher seroprevalence of HCV in Ghanaian prison inmates was probably due to prolonged incarceration beyond 36 months, the higher proportion of individuals with prior histories of intravenous drug use and homosexual orientation/MSM (a high-risk sexual behaviour) amongst Ghanaian prisoners, compared to the general Ghanaian population [22].

To our knowledge, data on the risk factors associated with the higher prevalence of HIV, HBV, HCV and syphilis infections among prison inmates and officers in Ghana are very scanty, and therefore discussion(s) about bloodborne and sexually transmitted infections within the prisons often requires extrapolation from data obtained in other countries. Assessment of the extent and sources of risk of these bloodborne infections for prison inmates and officers in Ghana will facilitate decision making about how to screen for these infections, prevent further spread of these infections and provide appropriate care to infected prison inmates and officers. The aim of this study was to determine the prevalence and associated risk factors for HIV, HBV, HCV and syphilis infections among a sample of prison inmates and officers stationed at eight of the ten regional central prisons in Ghana.

## Methods

### Study sites

The study was designed as a national multicenter cross-sectional study and was conducted between the months of May 2004 and December 2005 among a convenient sample of male and female prison inmates and officers stationed at eight of the ten regional central prisons in Ghana. Ghana is divided into ten administrative regions, and the largest prison in each of these regions is the regional central prison in each regional capital. All prison or correctional officers in Ghana are not engaged or employed in healthcare delivery (PONEIHD). All the healthcare needs of the inmates and officers of the Ghana Prison Service are provided by health care workers (HCWs) employed by the Ghana Ministry of Health who are stationed at the onsite prison clinic or the other tertiary public hospital upon referral by the onsite prison clinic. We enrolled inmates and officers from eight of these ten prisons, but could not enrol from the other two regional central prisons (Upper East Region and Upper West Region Central Prisons) as originally planned because of financial constraints. The study received ethical approval from both the Ethical & Protocol Review Committee of the University of Ghana Medical School, Accra, Ghana, and the Board of the Ghana Prison Service, Accra, Ghana.

### Study populations

The study was proposed to the entire population of prison inmates and officers after an explanation of the purpose of the study at a meeting organized for that purpose in each of these prisons. They were informed that the study was confidential and that the information provided would not affect their incarceration and employment status, respectively. A total of 1366 inmates and 445 officers consented and were enrolled in the study. Written informed consent was obtained from each participant, and the information regarding the protocol and informed consent was presented to each participant at an appropriate literacy level. No prison officer took part in the processes of obtaining consent and collecting data in this study. This was to allay fears of any possible victimization or security purpose of the study by ensuring that no one was coerced into taking part in any aspect of it. The study was conducted in a confidential manner and unique study-generated identifiers, not including the name of the participant, were used to link questionnaires and serum samples; and this information was only accessible to the principal investigator (AAA). All participants were provided with pre- and post-test counseling.

### Personal risk-factor assessment questionnaire

The personal risk-factor assessment questionnaire was initially pilot tested on a small sample of prison inmates and officers prior to the data collection. Sensitive personal risk assessment questions were addressed in a composite risk assessment question that incorporated less sensitive questions related to male-to-male sexual contact and illicit intravenous drug use. However, this less sensitive composite risk assessment question was left unanswered by all the prison officers in the pilot testing, despite our assurances of anonymity with study-generated identifiers (rather than their names), confidentiality and non-disclosure of results to their employer. Subsequent focus group discussion with the prison officers participating in the pilot testing revealed their discomfort with truthfully answering this question and their lingering concerns about possible employment victimization, despite our assurances. Therefore, the final questionnaire for the officers did not include the composite question relating to male-to-male sexual contact and illicit intravenous drug use, since we were of the view that this question would be left unanswered or not truthfully answered by the majority of the prison officers.

### Sample collection and serological analysis

Blood samples (about 6 ml) were collected in plain tubes from each consenting participants. Samples were centrifuged and the serum kept at -20°C until analysed. Sera were tested at the Virology Unit, Noguchi Memorial Institute for Medical Research, for the presence of antibodies to HIV 1 and 2 (ELISA and Western Blot; Abbot and Cambridge Biotech, respectively), antibodies to HCV (ELISA and Western Blot; Abbot), antibodies to *Treponema pallidum *[*Treponema pallidum *particle agglutination (TPPA) test; Serodia Fujirebio] and HBsAg (monoclonal, ELISA; Abbot), in accordance with the respective manufacturer's instructions. Repeatedly reactive specimens by HIV and HCV ELISA were assayed by Western blot.

### Statistical analysis

The Statistical Analysis System (SAS Institute, Cary, NC, USA) version 9.1 was used to complete all data analyses. Odd ratios (ORs) and their 95% confidence interval (95% CI) were used to compare proportions in independent groups of categorical sociodemographic, sexual and behavioural variables in univariate analysis. A P value of < 0.05 was considered significant. Multiple logistic regression models were developed to identify the independent risk factors associated with positive test results and 95% confidence intervals were calculated for adjusted ORs. The categories of variables were combined into only two to four categories to avoid large confidence intervals. The age categories were based on thirds of the age distribution of all the participating prison inmates and officers in the study. Marital status was categorized as married or not married (includes single, divorced or separated, and widowed), and educational level attained by the inmates was classified as illiterate or at least primary education. Time served (from the beginning of their current period of incarceration to the day of the survey) was categorized as below the median of 36 months or above the median of 36 months, and sexual orientation was classified as heterosexual or homosexual (includes both MSM/gays and lesbians). The duration of employment in the Ghana Prison Service was categorized as below the median of 10 years or above the median of 10 years. Finally, after incorporating the data from our previous report on the correlates of HCV infection in this same 1366 prisoners [22] with the data of this study, univariate and multivariate analyses were used to determine the associations between HIV, HBV, HCV and syphilis infections among the 1366 inmates and 445 officers in Ghana.

## Results

Between May 2004 and December 2005, 1366 inmates (17.9% of the total of 7652 eligible inmates in the eight prisons studied) and 445 prison officers not engaged in healthcare deliver (PONEIHD) [20.8% of the total of 2139 eligible prison officers stationed at the eight study sites] in Ghana participated in the study. Of the 1366 study inmates, 1247 (91.3 %) were males and the median age was 36.5 years (range 16–84). Of the 445 study officers, 278 (62.5%) were males and the median age was 38.1 years (range 25–59 years). The median duration of time served (from the beginning of their current period of incarceration to the day of the survey) was 36 months (range 3–127 months). The median duration of employment in the Ghana Prison Service (from the beginning of their employment to the day of the survey) was 10 years (range 1.5–27 years). The above stated median age, percentage of participation at each of the eight study sites, median duration of incarceration, and median duration of employment were not statistically significantly different between the male and female prison inmates and officers (data not shown). The above stated sex (male: female) ratio, median age, age range, median duration of incarceration, and median duration of employment of the study inmates and officers were similar to that of all the eligible inmates and officers in these eight prisons at the various periods of the study in each prison (Ghana Prisons Service, 2005) (data not shown).

Table 1 shows the ORs and corresponding 95% CIs for HIV and syphilis seropositivity, and positive HBsAg status according to the sociodemographic characteristics of the 1366 study prisoners on univariate analysis. Table 2 shows the ORs and corresponding 95% CIs for HIV, HCV and syphilis seropositivity, and positive HBsAg status according to the sociodemographic characteristics of the 445 study prison officers on univariate analysis. Table 3 shows the ORs and corresponding 95% CIs for HIV and syphilis seropositivity, and positive HBsAg status according to the sociodemographic characteristics of the 1366 study prisoners on univariate analysis. Table 4 shows the ORs and corresponding 95% CIs for HIV, HCV and syphilis seropositivity, and positive HBsAg status according to the sociodemographic characteristics of the 445 study prison officers on univariate analysis. Table 5 shows the ORs and corresponding 95% CIs for HIV and syphilis seropositivity, and positive HBsAg status according to the sexual and behavioural characteristics of the 1366 study prisoners on univariate analysis. The results of multivariate analysis confirmed that all the sociodemographic and sexual behavoiural characteristics that reached statistical significant in the univariate analysis presented in Tables 1, 2, 3, 4, 5 were independent determinants of the respective infections (data not shown).

**Table 1:** Odd ratios (ORs) and corresponding 95% confidence intervals (95% CIs) for HIV and syphilis seropositivity, and positive HBsAg status among the 1366 study inmates in eight regional central prisons in Ghana on univariate analysis

**Regional central prison**	**No. of inmates**		**HIV seropositive**		**HBV seropositive**		**Syphilis seropositive**	
	
	**Eligible**	**Enrolled (%)**	**No. (%)**	OR (95% CI)*	**No. (%)**	OR (95% CI)*	**No. (%)**	OR (95% CI)*
Ashanti region Kumasi	1370	145 (10.6)	5 (3.4)	7.8 (4.2–11.3)	28 (19.3)	1.5 (1.1–3.4)	40 (27.6)	5.7 (3.7–13.8)
Brong Ahafo region Sunyani	789	146 (18.5)	5 (3.4)	7.8 (4.2–11.3)	43 (29.5)	2.7 (2.0–7.6)	25 (17.1)	3.3 (2.4–9.5)
Central region Cape Coast	918	173 (18.8)	1 (0.6)	1.4 (0.9–2.6)	41 (23.7)	2.1 (1.8–5.3)	25 (14.5)	2.2 (1.9–5.6)
Eastern region Nsawam	1345	72 (5.4)	37 (51.4)	119.4 (87.2–131.7)	15 (20.8)	1.7 (1.3–3.9)	12 (16.7)	2.8 (2.2–8.1)
Greater Accra region James Fort & Camp	1497	209 (14.0)	17 (8.1)	18.5 (9.8–28.1)	34 (16.3)	1	19 (9.1)	1
Northern region Tamale	354	204 (57.6)	6 (2.9)	6.6 (3.4–9.9)	65 (31.9)	3.1 (2.3–8.9)	23 (11.3)	1.6 (1.2–3.7)
Volta region Ho	599	195 (32.6)	8 (4.1)	9.3 (5.5–13.7)	42 (21.5)	1.9 (1.5–4.5)	25 (12.8)	1.9 (1.4–4.2)
Western region Sekondi	780	222 (28.5)	1 (0.5)	1	81 (36.5)	3.7 (2.8–10.1)	57 (25.7)	4.5 (3.1–11.3)
Total	7652	1366 (17.9)	80 (5.9)		349 (25.5)		226 (16.5)	

*Adjusted for age.

**Table 2:** Odd ratios (ORs) and corresponding 95% confidence intervals (95% CIs) for HIV, HCV and syphilis seropositivity, and positive HBsAg status among 445 prison officers stationed at eight regional central prisons in Ghana on univariate analysis

**Regional central Prisons & Headquarters**	**No. of inmates**		**HIV seropositive**		**HBV seropositive**		**HCV seropositive**		**Syphilis seropositive**	
	
	**Eligible**	**Enrolled (%)**	**No. (%)**	*OR (95% CI)	**No. (%)**	*OR (95% CI)	**No. (%)**	*OR (95% CI)	**No. (%)**	*OR (95% CI)
Ashanti region Kumasi	344	59 (17.2)	5 (8.5)	4.9 (3.0–9.8)	9 (15.3)	9.7 (5.5–15.2)	7 (11.9)	1.5 (0.9–3.3)	8 (13.6)	7.4 (3.8–13.4)
Brong Ahafo region Sunyani	159	66 (41.5)	5 (7.6)	4.4 (2.9–8.9)	9 (13.6)	8.6 (5.1–14.5)	13 (19.7)	2.4 (1.8–5.9)	8 (12.1)	6.6(3.6–12.1)
Central region Cape Coast	428	33 (7.7)	2 (6.1)	3.5 (2.5–7.7)	6 (18.2)	11.5 (6.2–17.6)	14 (42.4)	5.2 (3.2–11.5)	1 (3.0)	1.6 (1.1–3.8)
Eastern region Nsawam	338	49 (14.5)	2 (4.1)	2.4 (1.3–5.5)	1 (2.0)	1.3 (0.8–2.7)	5 (10.2)	1	2 (4.1)	2.2 (1.7–5.7)
Greater Accra region James Fort & Camp	154	54 (35.1)	1 (1.9)	1	1 (1.9)	1	6 (11.1)	1.4 (0.8–2.9)	8 (14.8)	8.1 (4.3–14.9)
Northern region Tamale	128	67 (52.3)	3 (4.5)	2.6 (1.5–6.0)	10 (14.9)	9.4 ()	12 (17.9)	2.2 (1.6–5.4)	2 (3.0)	1.6 (1.1–3.8)
Volta region Ho	243	48 (19.8)	1 (2.1)	1.2 (0.7–2.3)	10 (20.8)	13.1 (7.3–22.4)	10 (20.8)	2.5 (1.9–6.2)	1 (2.1)	1
Western region Sekondi	345	69 (20.0)	3 (4.3)	2.5 (1.4–5.7)	6 (8.7)	5.5 (3.0–10.9)	16 (23.2)	2.8 (2.1–7.7)	5 (7.2)	3.9 (2.8–10.5)
Total	2139	445 (20.8)	22 (4.9)		52 (11.7)		83 (18.7)		35 (7.9)	

*Adjusted for age.

**Table 3:** Odd ratios (ORs) and corresponding 95% confidence intervals (95% CIs) for HIV and syphilis seropositivity, and positive HBsAg status according to the sociodemographic characteristics among 1366 prisoners in Ghana on univariate analysis

Sociodemographic characteristic*	**No. of inmates (%)**	**HIV seropositive**		**HBV seropositive**		**Syphilis seropositive**	
		
		**No. (%)**	**OR (95% CI)#**	**No. (%)**	**OR (95% CI)#**	**No. (%)**	**OR (95% CI)#**
**Age (years)**							
17–31	455 (33.3)	37 (8.1)	3.9 (2.6–9.8)	178 (39.1)	3.2 (2.4–8.5)	111 (24.4)	3.3 (2.5–8.9)
32–46	458 (33.5)	30 (6.6)	3.1 (2.2–7.7)	105 (22.9)	2.4 (1.7–6.3)	71 (15.5)	2.2 (1.4–6.1)
>47	453 (33.2)	13 (2.9)	1	66 (14.5)	1	44 (9.7)	1
**Marital status**							
Married	1030 (75.4)	34 (3.3)	1	197 (19.1)	1	102 (9.9)	1
Not married	336 (24.6)	46 (13.7)	5.9 (3.3–14.2)	152 (45.2)	2.9 (2.2–7.1)	124 (36.9)	4.9 (4.1–12.7)
**Educational level attained**							
Primary or better	1123 (82.2)	48 (4.3)	1	219 (19.5)	1	117 (10.4)	1
Illiterate or none	243 (17.8)	32 (13.2)	4.2 (2.8–11.0)	130 (53.5)	3.7 (2.7–10.6)	109 (44.9)	5.6 (3.1–13.6)
**Gender**							
Female	119 (8.7)	18 (15.1)	4.5 (3.0–13.4)	51 (42.9)	3.5 (2.5–9.8)	43 (36.1)	3.4 (2.3–8.5)
Male	1247 (91.3)	62 (5.0)	1	298 (23.9)	1	183 (14.7)	1

*Combined into only two to three categories to minimize the width of confidence intervals.

#Adjusted for age.

**Table 4:** Odd ratios (ORs) and corresponding 95% confidence intervals (95% CIs) for HIV, HCV and syphilis seropositivity, and positive HBsAg status according to the sociodemographic characteristics among 445 prison officers in Ghana on univariate analysis

Sociodemographic characteristic*	**No. of inmates (%)**	**HIV seropositive**		**HBV seropositive**		**HCV seropositive**		**Syphilis seropositive**	
		
		**No. (%)**	**OR (95% CI)#**	**No. (%)**	**OR (95% CI)#**	**No. (%)**	**OR 95% CI)#**	**No. (%)**	**OR (95% CI)#**
**Age (years)**									
25–35	149 (33.4)	8 (5.4)	2.2 (1.5–5.4)	22 (14.8)	2.4 (1.8–6.4)	29 (19.5)	2.3 (1.7–5.9)	14 (9.4)	2.0 (1.5–4.9)
36–46	148 (33.3)	10 (6.8)	2.8 (2.0–7.8)	19 (12.8)	2.1 (1.5–5.2)	38 (25.7)	3.0 (2.2–8.9)	13 (8.8)	1.9 (1.4–4.7)
>47	148 (33.3)	4 (2.7)	1	11 (7.4)	1	16 (10.8)	1	8 (5.4)	1
**Gender**									
Female	167 (37.5)	12 (7.2)	2.2 (1.5–5.4)	29 (17.4)	2.5 (1.8–6.7)	38 (22.8)	1.8 (1.3–4.5)	20 (12.0)	2.6 (1.9–7.0)
Male	278 (62.5)	10 (3.6)	1	23 (8.3)	1	45 (16.2)	1	15 (5.4)	1
**Marital status**									
Married	203 (45.6)	8 (3.9)	1	20 (9.9)	1	33 (16.3)	1	12 (5.9)	1
Not married	242 (54.4)	14 (5.8)	1.6 (1.1–3.4)	32 (13.2)	1.6 (1.2–3.5)	50 (20.7)	1.6 (1.2–3.6)	23 (9.5)	1.9 (1.4–4.7)
**Educational level attained**									
Middle School	144 (32.4)	9 (6.3)	1.5 (0.9–2.8)	20 (13.9)	1.4 (0.7–2.3)	30 (20.8)	1.3 (0.7–2.0)	14 (9.7)	1.5 (0.8–2.7)
Junior Secondary School	69 (15.5)	4 (5.8)	1.4 (0.9–2.6)	10 (14.5)	1.5 (0.9–2.8)	15 (21.7)	1.4 (0.8–2.3)	5 (7.2)	1.1 (0.5–1.8)
Senior Secondary School	212 (47.6)	8 (3.8)	1	21 (9.9)	1	34 (16.0)	1	14 (6.6)	1
University/College	20 (4.5)	1 (5.0)	1.3 (0.7–2.0)	2 (10.0)	1.0 (0.3–1.3)	4 (20.0)	1.3 (0.7–2.0)	2 (10.0)	1.5 (0.8–2.7)
**Duration of employment in prison service (years)**									
Below median (<10)	223 (50.1)	9 (4.0)	1	20 (9.0)	1	33 (14.8)	1	15 (6.7)	1
Above median (>10)	222 (49.9)	13 (5.9)	1.6 (1.1–3.4)	32 (14.4)	1.9 (1.4–4.5)	50 (22.5)	1.9 (1.4–4.6)	20 (9.0)	1.5 (1.1–3.2)
**History of STD**									
Yes	75 (16.9)	6 (8.0)	2.0 (1.4–4.7)	17 (22.7)	2.9 (2.1–8.2)	21 (28.0)	2.1 (1.5–5.3)	14 (18.7)	3.8 (2.6–10.5)
No	370 (83.1)	16 (4.3)	1	35 (9.5)	1	62 (16.8)	1	21 (5.7)	1
**History of blood transfusion**									
Yes	90 (20.2)	5 (5.6)	1.3 (0.7–2.0)	11 (12.2)	1.3 (0.6–2.0)	19 (21.1)	1.5 (0.9–2.8)	8 (8.9)	1.3 (0.6–2.0)
No	355 (79.8)	17 (4.8)	1	41 (11.6)	1	64 (18.0)	1	27 (7.6)	1
**Ever been tattooed or pierced**									
Yes	58 (13.0)	3 (5.2)	1.2 (0.6–1.9)	7 (12.1)	1.3 (0.6–2.0)	11 (19.0)	1.3 (0.7–2.0)	5 (8.6)	1.3 (0.6–2.0)
No	387 (87.0)	19 (4.9)	1	45 (11.6)	1	72 (18.6)	1	30 (7.8)	1
**Ever been cut or assaulted**									
Yes	93 (20.9)	5 (5.4)	1.2 (0.6–1.9)	11 (11.8)	1.2 (0.5–1.8)	18 (19.4)	1.3 (0.7–2.0)	8 (8.6)	1.3 (0.6–2.0)
No	352 (79.1)	17 (4.8)	1	41 (11.6)	1	65 (18.5)	1	27 (7.7)	1

*Combined into only two to four categories to minimize the width of confidence intervals.

#Adjusted for age.

**Table 5:** Odd ratios (ORs) and corresponding 95% confidence intervals (95% CIs) for HIV and syphilis seropositivity, and positive HBsAg status according to the sexual behavioural characteristics among 1366 prisoners in Ghana on univariate analysis

Sexual and behavioural characteristic*	**No. of inmates (%)**	**HIV seropositive**		**HBV seropositive**		**Syphilis seropositive**	
		
		**No. (%)**	**OR (95% CI)#**	**No. (%)**	**OR (95% CI)#**	**No. (%)**	**OR (95% CI)#**
**Time served (months)**							
Below median (<36)	682 (49.9)	15 (2.2)	1	59 (8.7)	1	36 (5.3)	1
Above median (>36)	684 (50.1)	65 (9.5)	6.3 (2.7–16.5)	290 (42.4)	7.1 (3.0–18.3)	190 (27.8)	7.6 (3.2–20.1)
**Sexual orientation**							
Heterosexual	963 (70.5)	44 (4.6)	1	187 (19.4)	1	132 (13.7)	1
Homosexual	403 (29.5)	36 (8.9)	2.7 (1.9–7.8)	162 (40.2)	2.9 (2.2–8.3)	94 (23.3)	2.4 (1.5–6.9)
**Ever injected drugs**							
Yes	481 (35.2)	55 (11.4)	5.7 (2.4–12.8)	237 (49.3)	5.4 (2.2–11.1)	152 (31.6)	5.3 (2.2–10.7)
No	885 (64.8)	25 (2.8)	1	112 (12.7)	1	74 (8.4)	1
**Ever shared needles or injection implements**							
Yes	977 (71.5)	62 (6.3)	2.0 (1.3–5.2)	269 (27.5)	1.9 (1.2–5.0)	174 (17.8)	1.9 (1.2–5.1)
No	389 (28.5)	18 (4.6)	1	80 (20.6)	1	52 (13.4)	1
**Ever paid or been paid for sex**							
Yes	73 (5.3)	8 (11.0)	2.8 (1.8–8.2)	27 (37.0)	2.1 (1.4–5.5)	19 (26.0)	2.3 (1.5–6.3)
No	1293 (94.7)	72 (5.6)	1	322 (24.9)	1	207 (16.0)	1
**History of STD**							
Yes	491 (35.9)	46 (9.4)	3.4 (2.0–9.3)	178 (36.3)	2.6 (1.8–7.4)	113 (23.0)	2.5 (1.6–7.1)
No	875 (64.1)	34 (3.9)	1	171 (19.5)	1	113 (12.9)	1
**History of blood transfusion**							
Yes	961 (70.4)	57 (5.9)	1.4 (0.8–2.7)	249 (25.9)	1.5 (0.9–2.9)	162 (16.9)	1.5 (0.8–2.8)
No	405 (29.6)	23 (5.7)	1	100 (24.7)	1	64 (15.8)	1
**Ever had sex with an injection drug user**							
Yes	136 (10.0)	9 (6.6)	1.6 (0.9–3.1)	35 (25.7)	1.3 (0.6–2.3)	24 (17.6)	1.4 (0.8–2.6)
No	1230 (90.0)	71 (5.8)	1	314 (25.5)	1	202 (16.4)	1
**Ever snorted drugs**							
Yes	598 (43.8)	37 (6.2)	1.4 (0.8–2.7)	155 (25.9)	1.3 (0.7–2.4)	101 (16.9)	1.3 (0.7–2.5)
No	768 (56.2)	43 (5.6)	1	194 (25.3)	1	125 (16.3)	1
**Ever been tattooed or pierced**							
Yes	219 (16.0)	13 (5.9)	1.2 (0.5–1.8)	56 (25.6)	1.1 (0.4–1.5)	37 (16.9)	1.2 (0.6–1.8)
No	1147 (84.0)	67 (5.8)	1	293 (25.5)	1	189 (16.5)	1
**Ever been cut or assaulted**							
Yes	423 (31.0)	25 (5.9)	1.2 (0.5–1.9)	109 (25.8)	1.2 (0.5–2.0)	71 (16.8)	1.1 (0.4–1.6)
No	943 (69.0)	55 (5.8)	1	240 (25.5)	1	155 (16.4)	1

*Combined into only two to three categories to minimize the width of confidence intervals.

#Adjusted for age.

Among the 1366 study inmates, HIV seropositivity was significantly associated with positive HBsAg status (OR 5.3; 95% CI 2.1–9.9), HCV seropositivity (OR 6.6; 95% CI 3.4–10.8) and syphilis seropositivity (OR 11.8; 95% CI 5.7–23.1). Positive HBsAg status was significantly associated with HIV seropositivity (OR 3.5; 95% CI 2.0–8.5), HCV seropositivity (OR 3.6; 95% CI 2.1–8.8) and syphilis seropositivity (OR 5.1; 95% CI 2.9–11.4). HCV seropositivity was significantly associated with HIV seropositivity (OR 4.5; 95% CI 3.0–11.2), positive HBsAg status (OR 4.0; 95% CI 2.4–9.8) and syphilis (OR 4.1; 95% CI 2.6–10.1) seropositivity. Finally, syphilis seropositivity was significantly associated with HIV seropositivity (OR 5.9; 95% CI 3.2–14.1), positive HBsAg status (OR 6.1; 95% CI 3.6–15.7) and HCV seropositivity (OR 3.7; 95% CI 2.3–9.4) [data not shown].

Among the 445 study officers not engaged in healthcare delivery (PONEIHD), HIV seropositivity was significantly associated with positive HBsAg status (OR 11.1; 95% CI 6.0–16.7), HCV seropositivity (OR 11.4; 95% CI 6.2–17.5) and syphilis seropositivity (OR 24.0; 95% CI 11.4–39.6). Positive HBsAg status was significantly associated with HIV seropositivity (OR 6.9; 95% CI 4.2–11.6), HCV seropositivity (OR 3.5; 95% CI 2.2–7.6) and syphilis seropositivity (OR 6.2; 95% CI 3.5–10.9). HCV seropositivity was significantly associated with HIV seropositivity (OR 5.3; 95% CI 2.8–9.9), positive HBsAg status (OR 3.2; 95% CI 2.1–7.2) and syphilis seropositivity (OR 3.8; 95% CI 2.3–8.2). Finally, syphilis seropositivity was significantly associated with HIV seropositivity (OR 14.6; 95% CI 8.3–26.4), positive HBsAg status (OR 7.4; 95% CI 3.9–13.0) and HCV seropositivity (OR 4.7; 95% CI 2.6–9.1) [data not shown].

## Discussion

Correctional facilities or prisons form part of the criminal justice system and it is estimated that over 9 million people are in penal institutions worldwide. Overcrowding in prisons remains a concern in both developed and developing countries, and is a key causative factor for a myriad of other problems which ultimately turn these custodial settings into fertile breeding grounds for infectious diseases such as AIDS, hepatitis, syphilis, gonorrhoea and tuberculosis. Compared to the general population, prisoners worldwide continue to demonstrate a significantly higher prevalence of bloodborne and sexually transmitted infections [1-8,19,23-31]. Prison officers not engaged in healthcare delivery (PONEIHD) [category II workers] are recognized to be at increased risk for these infections because of the high prevalence of these infections among inmates and their close interaction with the inmates in the performance of their job [32-34]. Knowledge of the correlates of bloodborne viral infections and sexually transmitted diseases (STDs) in different parts of the world, and particularly in Africa in the face of limited resources, is important for the planning of preventive measures and the development of vaccination programmes. Furthermore, the comparison of the prevalence among prison inmates, prison officers and the general population in the same geographical area is important to provide a basis for action, and changes in public health policy, education and clinical practice. Numerous studies have estimated the occupational risk of exposure and infection with bloodborne pathogens for hospital-based and correctional healthcare workers (category I workers) [9-14], but similar data are sparse for PONEIHD. Systematic surveillance of HIV, HBV, HCV and syphilis in prison inmates and officers in Ghana is completely absent, and there is no formal program for institutionalized implementation of standard infection control practices, bloodborne infectious disease risk reduction recommendations, mandatory screening and HBV immunization upon employment and treatment for both prison inmates and officers in the Ghana Prison Service.

This study found higher overall seroprevalence rates of HIV (5.9%), HBV (25.5%) and syphilis (16.5%) in Ghanaian prisoners, as compared to the seroprevalence of these infections previously reported in the general public in Ghana, mainly healthy blood donors and pregnant women [35-44]. The present study supports previous reports that prisoners represent a high-risk group for bloodborne diseases and STDs [1-8,19,23-31]. The overall seroprevalence of HIV, HBV and HCV [22] among Ghanaian prisoners (5.9%, 25.5% and 18.7%, respectively) is higher than the results of a similar study in incarcerated populations in the United Kingdom (0.4%, 8.0% and 7.0%, respectively) [25]. Additionally, the overall seroprevalence of HIV, HBV, syphilis and HCV [22] among Ghanaian prisoners (5.9%, 25.5%, 16.5% and 18.7%, respectively) is higher than the results of a similar study in incarcerated populations in Brazil (3.2%, 17.5%, 7.4% and 6.3%, respectively) [28]. Finally, the overall seroprevalence of HBV and syphilis among Ghanaian prisoners (25.5% and 16.5%, respectively) is higher than the results of a similar study in incarcerated populations in the United States of America (25.2% and 0.6%, respectively) [45], however that of HIV and HCV [22] in Ghanaian prisoners (5.9% and 18.7%, respectively) is lower than that in prisoners in the United States of America (6.6% and 29.7%, respectively) [45]. From the comparison above, the higher prevalence among Ghanaian prisoners compared to prisoners in the United States of America [45], the United Kingdom [25] and Brazil [28] most probably reflects the current absence of any harm reduction interventions in Ghanaian prisons whilst variable extent of implementation of such interventions are currently in place in prisons in the United States of America, the United Kingdom and Brazil. However, the higher prevalence of HIV and HCV in prisoners in the United States of America compared to Ghanaian prisoners (in whom no harm reduction interventions have yet been implemented) most probably reflects a persistently higher prevalence of intravenous drug use within and without the prisons in the United States of America compared to Ghana, despite the implementation of harm reduction interventions within most prisons in the United States of America.

This study found higher overall seroprevalence rates of HIV (4.9%), HBV (11.7%), HCV (18.7%) and syphilis (7.9%) in Ghanaian PONEIHD, as compared to the seroprevalence of these infections previously reported in the general public in Ghana, mainly healthy blood donors and pregnant women [35-44]. However, the seroprevalence in the prison officers is comparable but generally lower to the similarly high seroprevalence observed in prisoners in this study and previously reported in prisoners in Ghana (5.9%, 25.5%, 16.5% and 18.7%, respectively) [19], though the seroprevalence of HCV was even higher in the prison officers than the prisoners. Our observed overall HBsAg carrier rate of 11.7% among Ghanaian PONEIHD is higher than the results of a similar seroepidemiology study in PONEIHD in Italy (6.6%), which reported a comparable HBsAg carrier rate of 6.7% in prisoners in Italy [20]. However, our observed overall HBsAg carrier rate of 11.7% among Ghanaian PONEIHD is lower than that of a similar study in PONEIHD in Greece (20.0%), which was higher than the HBsAg carrier rate of 13.3% in prisoners in Greece [21]. Our observed HCV seroprevalence of 18.7% in Ghanaian PONEIHD is much higher than that reported in PONEIHD in Greece (5.0%), which was lower than the HCV seroprevalence of 15.5% in prisoners in Greece [21]. A more recent study among correctional healthcare workers or prison officers employed in healthcare delivery in three states in the United States of America reported no evidence of chronic HBV infection (ie, 0% HBsAg carrier rate) and 2% HCV seroprevalence [10], which was much lower that that herein reported for PONEIHD in Ghana and that previously reported for prison officers in Italy [20] and Greece [21]. From the comparison above, the higher seroprevalence of especially HCV among Ghanaian prison officers compared to prison officers in the United States of America [10], Italy [20] and Greece [21] most probably reflects the current absence of any institutionalized infection control measures and harm reduction interventions in Ghanaian prisons whilst variable extent of implementation of such interventions are currently in place in prisons in the United States of America, Italy and Greece.

The seroprevalence rates of HIV, HBV and syphilis infections were significantly higher in inmates aged 17–46 (which spans the generally accepted sexually-active age group of 15–45 years), unmarried inmates, illiterate inmates, female inmates, inmates incarcerated for longer than the median time served of 36 months, inmates reporting previous histories of homosexuality, of IDU, of sharing drug paraphernalia, or participating in paid sex, and of STD. These observed risk factors for all three infections in Ghanaian prisoners are generally considered as indicators of either intravenous drug use (history of IDU and sharing drug paraphernalia, and prolonged incarceration), high-risk sexual behaviour (sexually-active age group, unmarried, homosexual orientation, participation in paid sex and history of STD), or lower socioeconomic status (illiterate and female gender). The seroprevalence rates of HIV, HBV, HCV and syphilis infections were significantly higher in prison officers aged 25–46 (which spans the generally accepted sexually-active age group of 15–45 years), unmarried prison officers, female prison officers, prison officers employed for longer than the median duration of 10 years, and reporting a previous history of STD. These observed risk factors for all four infections in Ghanaian prison officers are related to the duration of exposure to work in the prison environment, high-risk sexual behaviour (sexually-active age group, unmarried, and history of STD), or lower socioeconomic status (female gender). Our observed significant associations between HIV, HBV, HCV and syphilis seropositivity status among the prison inmates and officers in Ghana suggests that the transmission of all four infections may be linked and are related to the same identified risk factors above.

The herein reported seroprevalence rates of HIV, HBV, HCV and syphilis are more representative of the Ghanaian prison population, because of the much larger sample size, than that previously reported [19]. The explanations for the varied seroprevalence of the four infections between the eight sites studied, and the variation in level of participation between the eight study sites are not obvious from this study. The significantly higher prevalence of these bloodborne and sexually transmitted infections in female prison inmates and officers compared to their male counterparts in Ghana is not unexpected, since similar gender disparity has been reported in prisoners and minority women in the southern United States of America [46-50] driven by similar predisposing sexual and socioeconomic risk factors as we have described above in prisons in Ghana. Addtionally, women are also more likely to engage in commercial sex work (CSW), a high-risk sexual behaviour that correlate with the prevalence of syphilis and other STDs [29,51-53]. Our observed gender disparity in the risk of these infections in the Ghanaian prison system most probably also reflects the general lower socioeconomic status of women worldwide (but especially in developing countries like Ghana), and emphasizes the specific needs of women in the implementation of efforts at reducing the risk for bloodborne and sexually transmitted infections.

The results of this study suggest that PONEIHD in Ghana may have exposure rates to bloodborne infections that are comparable to those in published reports regarding hospital-based health care workers (HCWs) [category I exposure risk rather than category II exposure risk) [9,11,14]. This suggested level of exposure is surprising, since PONEIHD in Ghana do not administer any acute healthcare to prisoners compared to that done by HCWs in acute care hospitals. Apart from occupational exposure, the exchange of sexual favours between prison officers and inmates, which is not unheard of, may be another mitigating factor in the comparably high seroprevalence of these bloodborne and sexually transmitted infections in Ghanaian prison inmates and officers. A concerning finding was that the prevalence of HCV infection in the prison officers was even higher than that in prisoners in this study and previously reported in prisoners in Ghana [19]. Therefore, the present study substantiates the concern that PONEIHD may be at significantly increased risk of bloodborne infections, especially HCV, because of the particularly high prevalence of HCV among prisoners worldwide [1-8,19,23-31]. The observation of the comparably higher prevalence of antibodies to these infections among the prison officers and prisoners, compared to the general Ghanaian population, is strong presumptive evidence of intra-prison transmission between prisoners and prison officers. The cross-sectional scope of the current study did not allow us to determine whether the prison inmates and officers acquired the diseases while within or outside of the prison. However, growing evidence also suggests that in general, prisoners are more sexually active in the community than the general population, reporting a higher number of partners and lower use of condoms [28,30]. Most prisoners have the high-risk behaviours for the transmission of these infections. Besides illicit drug use, unsafe sex and homosexuality, prisoners frequently tattoo their skins out of boredom, and in the process share needles and ink. These high-risk behaviours place prisoners at increased risk of infection with blood-borne viruses in comparison to the rest of the population [22,28,30].

The cross-sectional study design, reliance on self-reporting, sociocultural and religious beliefs, and job security/victimization concerns of the study participants (despite all our assurances of confidentiality during the explanation of the purpose of the study) may be possible biases and limitations of the study. In spite of these possible biases or limitations, this study provides important information previously unavailable on prisoners and PONEIHD in Ghana and can serve as a platform on which other studies can build. This study would contribute to efforts to respond to the important healthcare needs of these vulnerable individuals in Ghana. The results of the study reveal worrying trends for officials of the Ghana Prisons Service and the Ministries of Interior and Health, Accra, Ghana, who are charged with the management of bloodborne and sexually transmitted infections in the country. The implications of these findings for HIV, HBV, HCV and syphilis public health education are obvious. The results reported herein have significant implications for penal and public health officials, and suggest the urgent need for the introduction of policies to prevent the transmission of these infections in the prison environment. Even with resource limitations in Africa, effective partnerships between the prison officials, governmental, non-governmental and donor organizations would make headway in addressing issues of prison health as public health issues in Africa. The development and implementation of effective prison health initiatives providing counseling, health education, confidential voluntary testing, care pathways, strategies for interrupting transmission as well as harm reduction in Ghana is urgent and long overdue. Such initiatives would ultimately benefit the inmates, the correctional officers, families, communities and the general Ghanaian population.

The main focus of these risk reduction efforts should be on providing access to the prison environment to skilled infection control practitioners to create a strong safety climate that is supportive of safe work practices, the use of safety equipments and other engineering controls, and urgent implementation and compliance with vaccine recommendations, especially for HBV. This has to be a nationwide effort, with support for infection control coming from the highest possible political and administrative level. A clear, thoughtful, well-planned, and carefully structured risk reduction approach that targets specific corrections-related barriers to infection control is urgently needed for prison officers in Ghana. Additionally, postexposure management protocols should be in place, and prison officers should be well informed about the procedures they should follow in the event of percutaneous injuries whilst on the job. This training should be documented, and employee vaccination records should be well maintained. We also recommend that all prison officers must be screened at the time of employment to determine their status with regards to these bloodborne and sexually transmitted infections, and HBV vaccination offered to seronegative new hires. Although, the prison inmates and officers participating in our study cannot be considered representative of all the prison inmates and officers in Ghana, the results reported herein have significant implications for penal and public health officials, suggesting the importance of introducing policies to prevent transmission of these infections within the prison system. These policies must include testing programmes in prisons, which should be seen as an opportunity to improve the health status of the infected prison inmates and officers, and prevent further transmission of the infectious agents, within and without the prisons. Finally, we recommend that the prison population (both prison officers and prisoners) should be included in the routine national disease surveillance programs of the Ministry of Health so as to provide a more representative picture of the epidemiology of bloodborne and sexually transmitted infections in Ghana.

The policy of the Ghana Prison Service is to send prisoners to any prison in Ghana where there is a vacancy at the time of committal, and not necessarily to the prison nearest to the site of crime or the site of trial. Additionally, the policy of the Ghana Prison Service is to post prison officers based on rank, expertise and qualification to any prison in Ghana where there is a vacancy. Hence, the absence of these two regional central prisons is not likely to impact the representativeness of our sample. However, the sociocultural and religious beliefs, and security concerns of the study participants may be additional limitations of the study, despite all our assurances of confidentiality during the explanation of the purpose of the study. As a result, some inmates may not have responded correctly to the parts of the questionnaire relating to sexual orientation, drug use and history of STD. In spite of these limitations, the study results provide important data on the correlates of HIV, HBV and syphilis infections among people sent to jail, and may contribute to efforts to respond to the important healthcare needs of these vulnerable individuals in Ghana. These policy strategies must include increasing prisoner and correctional staff education about the prevention of these infections, testing for and treatment of infected inmates, education in tattoo application and the provision of professional tattooing services, the provision of bleach, and the provision of clean injecting equipment [54]. The implementation of these harm reduction programme in prisoners in Ghana should be seen as an opportunity to improve the health status of the infected prison inmates and to prevent further transmission of these infections, within and without the prisons. As a rule, there are inadequate medical facilities and staff in the prisons in Ghana, and access to appropriate care outside the Ghana prison system is very difficult for the inmates. Although the World Health Organization's (WHO) guidelines for HIV/AIDS in prisons stipulate that all inmates have a right to equitable health care and that national AIDS programmes should be applied in jails, this is rarely the case in many African countries, including Ghana. Ultimately, this approach benefits not only prisoners but also prison staff and the public, and does not entail lessening of the safety and security of prisons. Most inmates eventually get released and those infected represent a serious risk to their families and communities as they are reservoirs for further spread in the general population. Further studies are underway to determine the modes of transmission, and to institute interventional measures.

## Conclusion

Intravenous drug use, high-risk sexual behaviours, and low socioeconomic status have resulted in the concentration of a population infected with HIV, HBV and syphilis in Ghanaian prisons. Duration of employment in the prison service, lower socioeconomic status of women, and high-risk sexual behaviour have resulted in a higher prevalence of HIV, HBV, HCV and syphilis in Ghanaian prison officers, than the general Ghanaian population. The high prevalence of these bloodborne and sexually transmitted infections found in Ghanaian prison inmates and officers suggests the urgent need for the introduction of some of the range of effective preventive strategies employed in prisons elsewhere. Despite current financial and institutional constraints, collaborations have to be developed between the Ghana Prisons Service, local government authorities, academic and research institutions, non-governmental agencies, community-based organizations, and donor agencies (both local and international) to provide effective prison health initiatives and harm reduction services within the prisons.

## List of abbreviations

AIDS, acquired immunodeficiency syndrome; CSW, commercial sex work/worker; HBsAg, surface antigen of hepatitis B virus; HBV, hepatitis B virus; HCV, hepatitis C virus; HCW, health-care workers, HIV, human immunodeficiency virus; IDU, injection drug use/user; MSM, men who have sex with men; OR, odds ratio; PONEIHD, prison officers not employed in healthcare delivery, STD, sexually transmitted disease; WHO, World Health Organization; 95% CI, 95% confidence interval

## Competing interests

The author(s) declare that they have no competing interests.

## Authors' contributions

AAA conceived the study, provided guidance to all aspects of this study, and revised the manuscript for important intellectual content. AAA, HBA, FG, WKA, IB, CA, IA, IFAH and GM participated in the design and coordination of the study, data and sample collection, and performed and supervised the immunoassays. HBA and GM performed the quality assessment of the data, data analysis, and data preparation. HBA drafted the manuscript. All authors read and approved the final manuscript.

## Pre-publication history

The pre-publication history for this paper can be accessed here:


